# Synthesis of MnCo_2_O_4_ nanoparticles as modifiers for simultaneous determination of Pb(II) and Cd(II)

**DOI:** 10.1371/journal.pone.0210904

**Published:** 2019-02-06

**Authors:** Vesna Antunović, Marija Ilić, Rada Baošić, Dijana Jelić, Aleksandar Lolić

**Affiliations:** 1 Faculty of Medicine, University of Banja Luka, Banja Luka, Bosnia and Herzegovina; 2 University of Belgrade—Faculty of Mining and Geology, Belgrade, Serbia; 3 Department of Analytical Chemistry, University of Belgrade—Faculty of Chemistry, Belgrade, Serbia; Institute of Materials Science, GERMANY

## Abstract

The porous spinel oxide nanoparticles, MnCo_2_O_4_, were synthesized by citrate gel combustion technique. Morphology, crystallinity and Co/Mn content of modified electrode was characterized and determined by Fourier transform infra-red spectroscopy (FT-IR), scanning electron microscopy (SEM), energy dispersive spectrometry (EDS), X-ray diffraction pattern analysis (XRD), simultaneous thermogravimetry and differential thermal analysis (TG/DTA). Nanoparticles were used for modification of glassy carbon electrode (GCE) and new sensor was applied for simultaneous determination of Pb(II) and Cd(II) ions in water samples with the linear sweep anodic stripping voltammetry (LSASV).The factors such as pH, deposition potential and deposition time are optimized. Under optimal conditions the wide linear concentration range from 0.05 to 40 μmol/dm^3^was obtained for Pb(II), with limit of detection (LOD) of 8.06 nmol/dm^3^ and two linear concentration ranges were obtained for Cd(II), from 0.05 to 1.6 μmol/dm^3^ and from 1.6 to 40 μmol/dm^3^, with calculated LOD of 7.02 nmol/dm^3^. The selectivity of the new sensor was investigated in the presence of interfering ions. The sensor is stable and it gave reproducible results. The new sensor was succesfully applied on determination of heavy metals in natural waters.

## Introduction

Spinel structures with general formula AB_2_O_4_ are well known materials in the field of catalysis, especially in the area of neutralizing the harmful components from environment, such as heavy metals [1,2]. Contamination of heavy metal ions is one of the most serious environmental issues, since heavy metal ions are not biodegradable and they accumulate in living organisms, tracking the heavy metal concentration in environment is of utmost importance. Heavy metal content is often quantified by well-known analytical methods, such as atomic absorption spectrometry (AAS) [3,4] inductively coupled plasma-mass spectrometry (ICP-MS) [5,6], inductively coupled plasma-optical emission spectrometry (ICP-OES) [7,8]and X-ray fluorescence spectrometry (XRF) [9]. Besides numbered techniques, electrochemical methods are also often used in analysis of heavy metals, mostly due to their high sensitivity, easy operation and transferability [10–12]. Modification of electrode surface by nanostructures such as carbon nanotubes (CNT), graphene or nanoparticles is one of the strategies for surface modification. Numerous papers reported electrode modification such as Cu-CeO_2_ coated with multiwall carbon nanotubes for determination of guanine and adenine [13] or Fe_3_O_4_/RGO nanoparticles for glassy carbon electrode modification for detection of Cd(II) ions [14]. Determination of various ions such as Zn(II), Cd(II), Pb(II), Cu(II) and Ag(I) using electrode modified with chromium oxide were reported [11].ZnO are often used as sensing devices in chemical, pharmaceutical and/or agricultural applications [15,16], the group of authors investigated the application of graphene nanoparticles on environmental pollutants and detection of Al(III) ions [17,18].

Present paper focus on spinel structure material that consist of Mn(II) and Co(II) combined in formula MnCo_2_O_4_, where Mn(II) occupies tetrahedral and Co(II) octahedral sites of crystal structure and its application for catching Cd(II) and Pb(II) ions in water samples. Spinel structure containing 3d elements, such as Mn and Co, or Fe, Cr and Ni have high spin electron configuration, due to oxygen ions being a weak field ligand. A great potential of MnCo_2_O_4_ in electrocatalysis [19] and in efficiency in polymer degradation [20] was reported. In order to make a good material with all good and desirable physicochemical characteristics, method of synthesis is very important. There are numerous papers concerning methods of synthesis of different spinel nanostructures, for electrochemical purpose, such as co-precipitation [21,22], sol–gel method [23,24], microemulsion method [25], hydrothermal [26], spray pyrolysis [27]. For Mn-Co spinel structure the most implemented methods are precipitation [1,28,29], hydrothermal method [30,31], combustion synthesis [32], or impregnation reaction [33]. It should be mentioned here that choice of precursor is also very important, since using hydroxides in precipitation reaction produce small surface area and possibility of shrinking the active surface very quickly [34]. The use of individual transition metals proved to be very good choice for synthesis of materials with specific surface area up to 100 m^2^/g which are thermally stable up to 500°C. Several precursors containing Mn and Co ions were used for spinel structure preparation such as: citrates [35], acetates [36], hydroxycarbonates [37], carbonates [35] and chlorides [1,29]. In this paper MnCo_2_O_4_ was synthesized by citrate-gel combustion technique. This sol-gel auto combustion technique is quite common, provides a very good homogeneity of samples, very easy control of stoichiometry and production at low cost. The proposed method involves metal salt (oxidizer) and organic complexant (reductant). Different salts can be used as precursors for auto-combustion technique but nitrates are the most commonly used. Furthermore, nitrates are hygroscopic and they absorb moisture well [38,39]. Citrate acid was used here as a fuel (reductant) in synthesis. Other chemicals, such as urea or glycin can also be used as a reductant. Glycine as as fuel was used for combustion synthesis of mesoporous MnO_2_/MnCo_2_O_4_ composite [32]. The choice of fuel, as well as the ratio of oxidizer/fuel affects morphology and electrochemical properties of electrode material [32,38].

Auto-combustion method is well known for production of nanodispersed simple or complex oxide, catalyst, superconductors etc. This synthesis method is quite common for synthesis of spinel-type ferrite nanomaterials [38], but according to authors' knowledge, there are no previously published papers describing the synthesis of MnCo_2_O_4_ nanoparticles by citrate-gel combustion technique for simultaneous determination of Cd(II) and Pb(II).

## Materials and methods

### Chemicals

Manganese nitrate, cobalt nitrate, 25% ammonium hydroxide and citric acid were obtained from Carlo Erba (Val de Ruil, France). Sulfuric acid (98%), acetic acid (98%), sodium acetate and potassium chloride were obtained from Betahem (Belgrade, Serbia). Working Pb(II) and Cd(II) were prepared from stock solutions used for atomic absorption (1000 ppm each, Merck). All reagents were analytical grade quality and used without further purification. Ultrapure water (Mili-Q plus 185, system Milipore) was used for preparation of all solutions.

### Preparation of supporting electrolytes

The solutions used as supporting electrolytes were prepared as follows: The H_2_SO_4_/KCl buffer solution was prepared by mixing equal volumes of the 20 mmol/dm^3^ sulfuric acid and 30 mmol/dm^3^ potassium chloride for pH 2. The acetate buffer solutions with different value from pH 4–6, were prepared by mixing appropriate volumes of 0.1 mol/dm^3^ acetic acid and 0.1 mol/dm^3^ sodium-acetate solutions.

### Instrumentation

Fourier transform infra-red spectroscopy (FT-IR) was carried out by using Tensor 27 instrument with addition of platinum stand on which a very small amount of spinel oxides sample was placed (Bruker, USA). The particle size and morphology of MnCo_2_O_4_ powder was observed by scanninig electron microscope (SEM) JEOL JSM-6390 LV. The X-ray diffractograms of the MnCo_2_O_4_ samples were obtained by means of Philips PW-1050 automatic diffractometer with a Cu K□1,2 line of 0.15418 nm. All electrochemical measurements were performed using a CHI 800C workstation (CH Instruments, USA). A three electrode system consisted of the glassy carbon working electrode (GCE; bare or modified), (CH instruments, USA; model CHI104), 3 mm in diameter. As reference electrode was used Ag/AgCl (CH Instruments, USA; CHI111) and platinum wire as auxiliary electrode.

### Synthesis of MnCo_2_O_4_spinel oxide nanoparticles

Synthesis of MnCo_2_O_4_ spinel oxide nanoparticles was carried out by citrate-gel combustion method. Manganese nitrate and cobalt nitrate solutions were mixed in molar ratio 1:2. Citric acid was used as a fuel. The mole ratio of citric acid versus nitrate groups was 1:3.6 as published elsewhere [40]. The pH 7 is adjusted by adding a solution of ammonium hydroxide. Solution was heated in an open glass beaker at temperature around 80°C under constant stirring (100 rpm) until light pink sol was formed. The sol turn into gel and it was finally calcinated for 2h at 450°C. The combustion of citric acid is presented by following equation (Eq 1):

$$2C_{6}H_{8}O_{7}+9O_{2}\to 12CO_{2}+8H_{2}O$$ (1)

Since the combustion temperature needed to complete combustion of remaining carbon residues is unknown, the heating at a constant temperature of 500°C was prolonged for approximately half an hour, which was enough to obtain carbon-free oxides. After calcination, a black powder of MnCo_2_O_4_ nanoparticles (NPs) was obtained.

### Preparation of glassy carbon electrode modified with MnCo_2_O_4_nanoparticles

Before modification, working electrode was pre-cleaned on a polishing pad using alumina slurry with different grain sizes (1, 0.3, and 0.05 μm, Buehler, USA). The electrode was rinsed with Mili Q water and methanol and ultrasonically dispersed for 3 min in a mixture of methanol and water (1:1, v/v). Suspension for modification was made by adding 1 mg of MnCo_2_O_4_ powder in 2 cm^3^ of pure water followed by sonication for 30 min. A 6 μL of the MnCo_2_O_4_ suspension was injected onto glassy carbon (GCE) mirror clean surface allowing the water evaporate at room temperature. The modified electrode is denoted as GCE-MnCo_2_O_4_NPs.

### Analytical procedure

The electrochemical measurements were performed in a three-electrode cell with a glassy carbon (bare or modified) electrode as the working electrode, a Ag/AgCl as the reference electrode, and a platinum wire as an auxiliary electrode. For pre-concentration step, the GCE-MnCo_2_O_4_NPs modified electrode was immersed into a H_2_SO_4_/KCl supporting electrolyte containing Pb(II) and Cd(II), and the accumulation and reduction of metal ions into metal (M^2+^ to M^0^) was performed under a constant potential at -1.4 V for 10 minutes with stirring. The deposition process was followed by the opposite process: re-oxidation of metals (M^0^) and stripping into solution metal ions (M^2+^). The electrochemical response was measured by linear sweep anodic stripping voltammetry (LSASV) in the potential range from -1.0 to 0 V vs. Ag/AgCl electrode with the scan in the anodic direction (re-oxidation of metal M^0^ to M^2+^). All electrochemical measurements are performed in triplicate (unless otherwise stated) and numbers are average values with apropriate confidence interval.

### Preparation of water samples

The application of the proposed sensor was performed by measuring the content of Pb(II) and Cd(II) in water samples. We used three different water samples: distilled water, tap water, and river water. River water samples were collected from Danube river (44°49′49″N and 20°27′47.5″E) on its right bank, closer to the Belgrade city centre, and tap water samples were collected from inside the faculty building (University of Belgrade, Faculty of Chemistry). Distilled and tap water samples were used without any prior preparation, while the sample of river water was filtered (membrane filter pore size 0.45 μm). Samples were prepared as follows: the appropriate volumes of the standard Pb(II) and Cd(II) solutions were transferred to a volumetric flask containing 2 cm^3^ of a water sample and 8 cm^3^ of supporting electrolyte. To remove dissolved oxygen through the prepared solutions, pure nitrogen was passed. Every sample was prepared in triplicate.

## Results and discussion

### Characterization of the MnCo_2_O_4_spinel material

**Fig 1:**
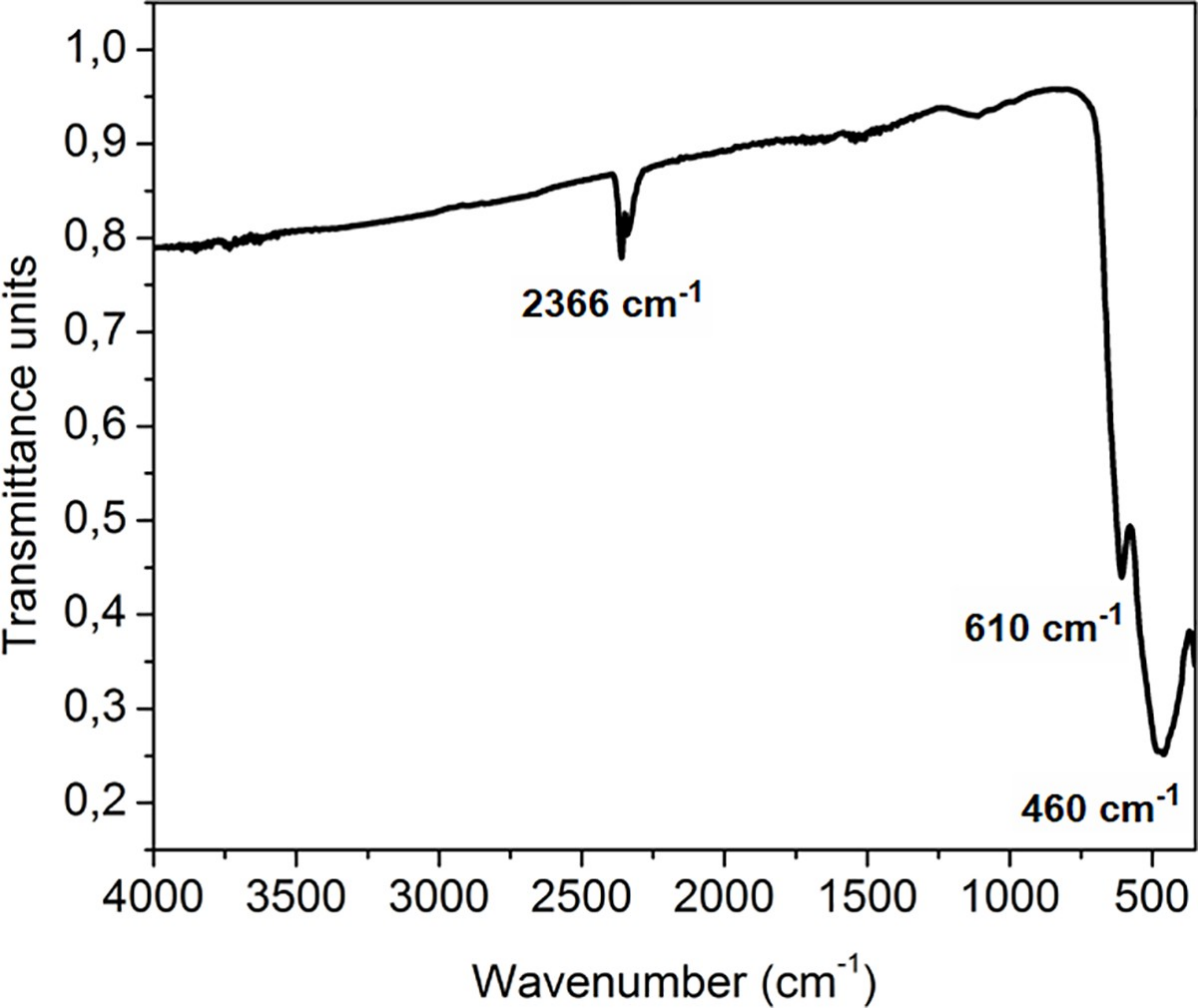
FTIR spectrum of MnCO_2_O_4_ nanoparticles in the spectrum range from 400 to 4000 cm^-1^.

FT-IR spectrum was observed in the range of 4000–400 cm^-1^ (Fig 1) which is usual for ion vibrations in the crystal lattice. The most prominent absorption bands at 610 cm^-1^ and 460 cm^-1^ are corresponding to metal-oxygen bonds in spinel structure of composite. Similar reports were for absorption peaks appearing in the range of 569–616 cm^-1^ represent high frequency bands which are characteristic of metal-oxygen vibrations in tetrahedral sites, while absorption peaks in the range of 426–471 cm^-1^ represent low frequency bands and this is caused by vibration of octahedral sites metal-oxygen bond [41]. Due to high temperature during the combustion process hydroxyl and nitrate groups are eliminated fromspinel material. The FTIR image also shows absorption band at ~2400 cm^-1^ which probably originates from adsorbed water molecules on the surface of MnCo_2_O_4_ nanoparticles [42,43].

**Fig 2:**
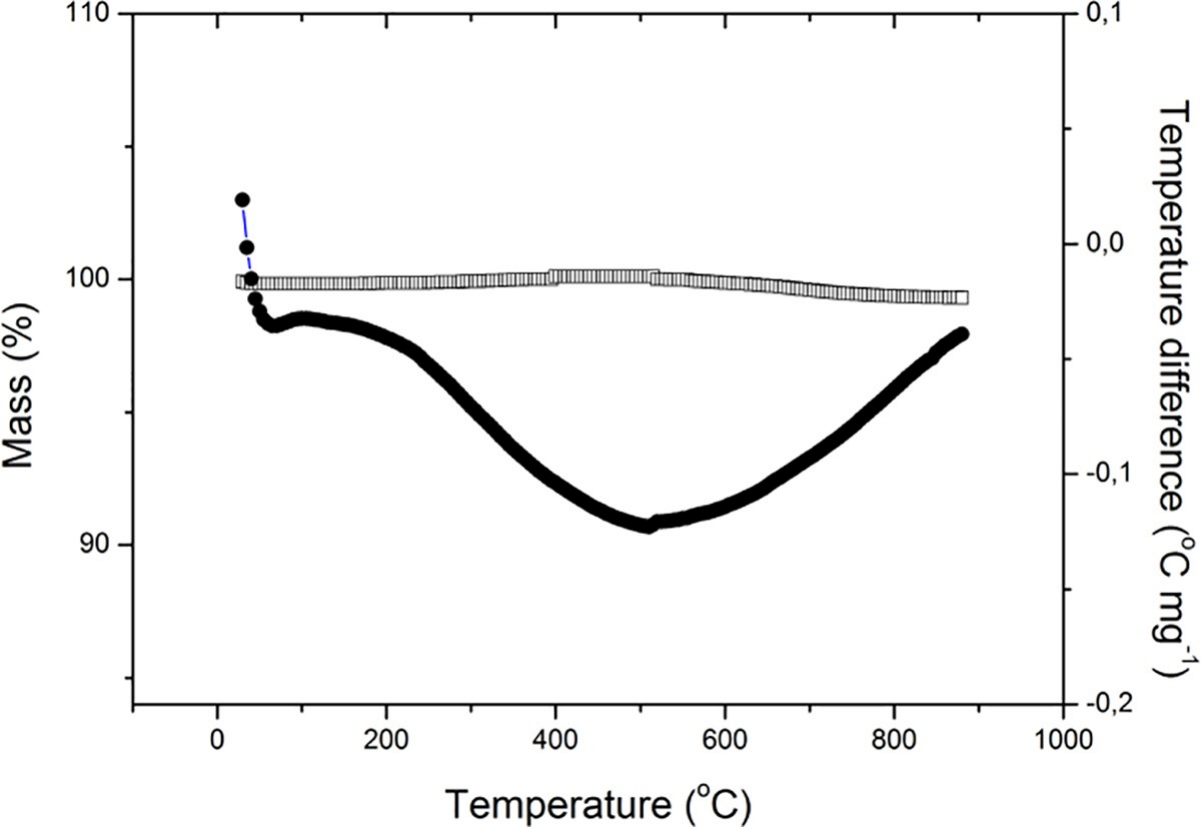
TG/DTA analysis of MnCo_2_O_4_ nanoparticles.

Presence of water was also detected by simultaneous TG/DTA analysis around 100°C presented with diffuse endothermic peak. Thermal analysis showed a very good thermal stability of prepared material in the temperature range from ambient up to 950°C, since mass loss presented in TG curve is quite insignificant and gradual disintegration of MnCo_2_O_4_ was not observed (Fig 2).

**Fig 3:**
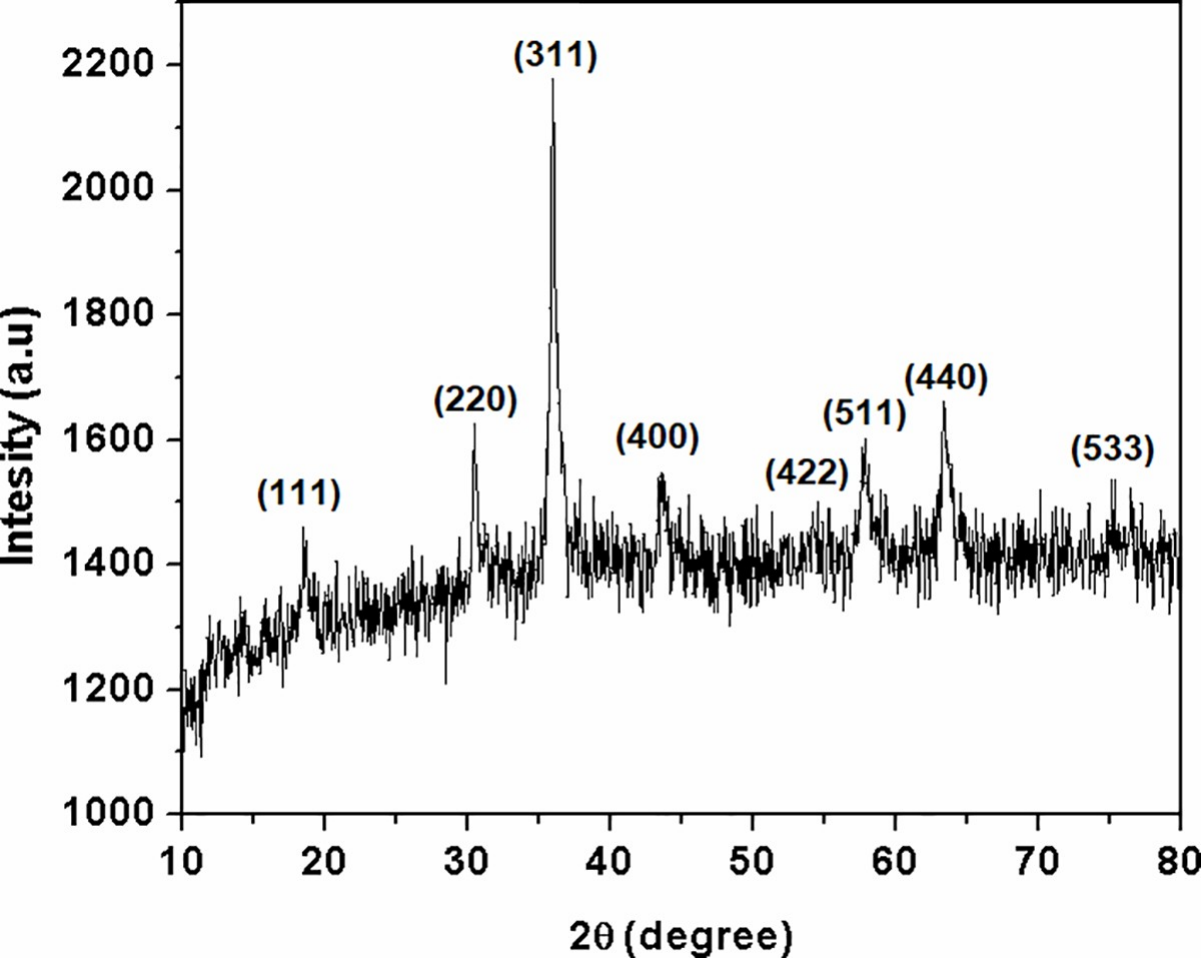
XRD pattern of 0.5MnO-0.5Co_2_O_4_ oxide system synthesized by citrate-gel combustion technique. The diagram indicates poorly crystallized (semiamorphous) MnCo_2_O_4_.

A representative X-ray diffraction patterns of the as-prepared final product is shown in Fig 3. Obtained X-ray spectrum shows to be in consistent with standard pattern of spinel structure of cubic MnCo_2_O_4_ with theoretical density of 5.564 g/cm (JCPDS card No. 23–1237) regarding on some diffraction peak positions. The results were consistent with results previously reported [42,44,45]. Diffraction peaks are corresponding to the Miller indices (111), (220), (311), (400), (422), (511), (440) and (533) revealed face-centered cubic spinel structure of the MnCo_2_O_4_[43,45]The most intense lines at 2θ of 18.53, 30.47, 35.99, 43.64, 54.30, 57.84, 63.45 degrees in XRD pattern are in good agreement with the angles (18.55, 30.54, 35.995, 43.759, 54.336, 57.909, and 63.622) in JCPDS card of MnCo_2_O_4_. As-prepared MnCo_2_O_4_ shows some nosier XRD spectrum than standard pattern of spinel structure of MnCo_2_O_4_ which is probably caused by low crystallite size [1,42]. In this case, Debye-Scherrer formula (Eq 2) based on full width at half maximum intensity of the (311) peak of the as-prepared MnCo_2_O_4_ can be used to calculate the average crystallite size which was amount to 19 nm.

D=0.9α/(βcosθ). (2)

**Fig 4:**
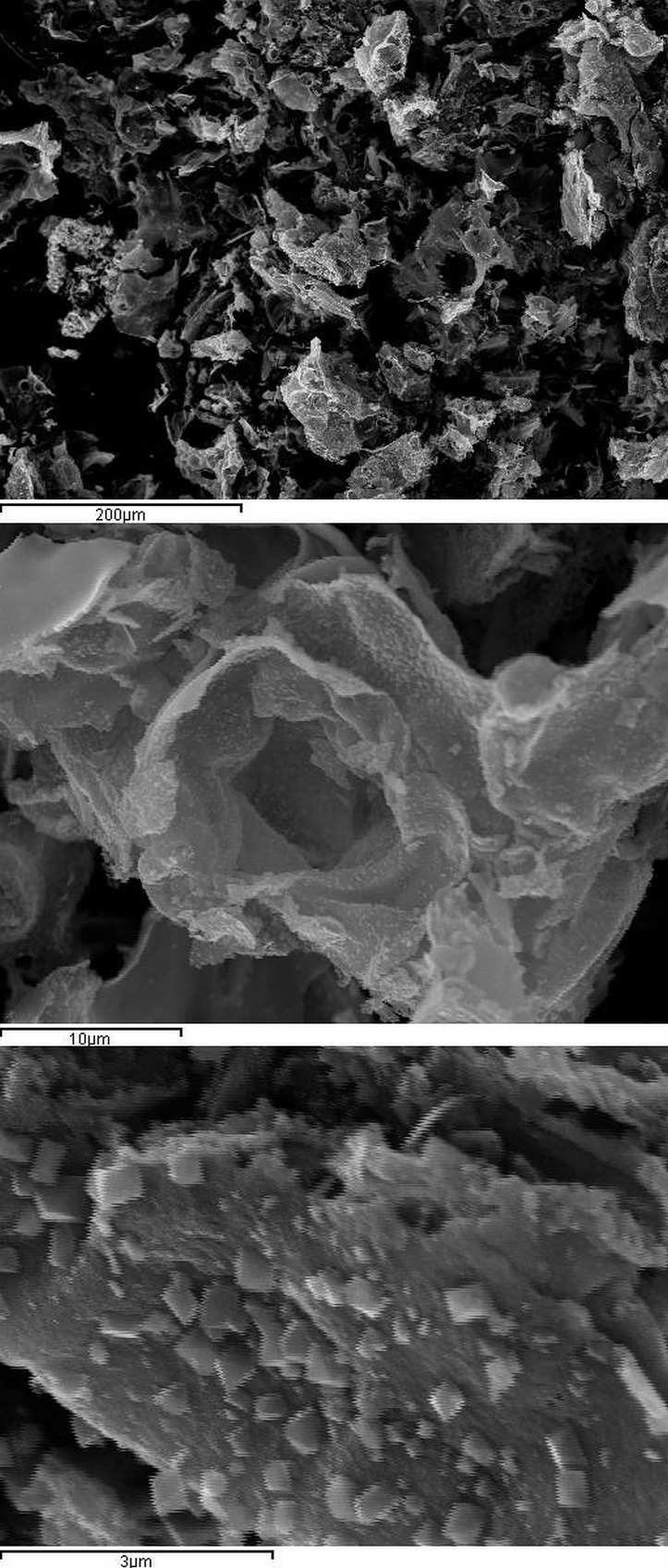
The SEM microphotographs of MnCo_2_O_4_ samples obtained with the temperature Tf = 500°C.

SEM images (Fig 4) show porous morphology of MnCo_2_O_4_. Flower-like structure is pronounced on the micrographs. Different polygonal particles are spread over the flower surface. Some cracks and defects are also visible. Morphological structure contains pores which probably allow transport of electrolyte, providing a good surface area for charge transfer reactions. This kind of ultrathin flow-like morphologies posses high electrochemical capacity which is responsible for efficient transport of electrons and ions [42,46]. As mentioned before this way of synthesis provides a good homogeneity of the nanocomposite which is mostly achieved with 1 to 1 molar ratio of the components [40]Assuming an almost spherical shape and accepting the mean particle radius r = 19 nm, the specific surface area was estimated on the basis of equation [47,48]

S=3/rd. (3)

Where d is the bulk density 5.564 g/cm was used. This calculation gave us the value of 28.37 m^2^/g.

**Fig 5:**
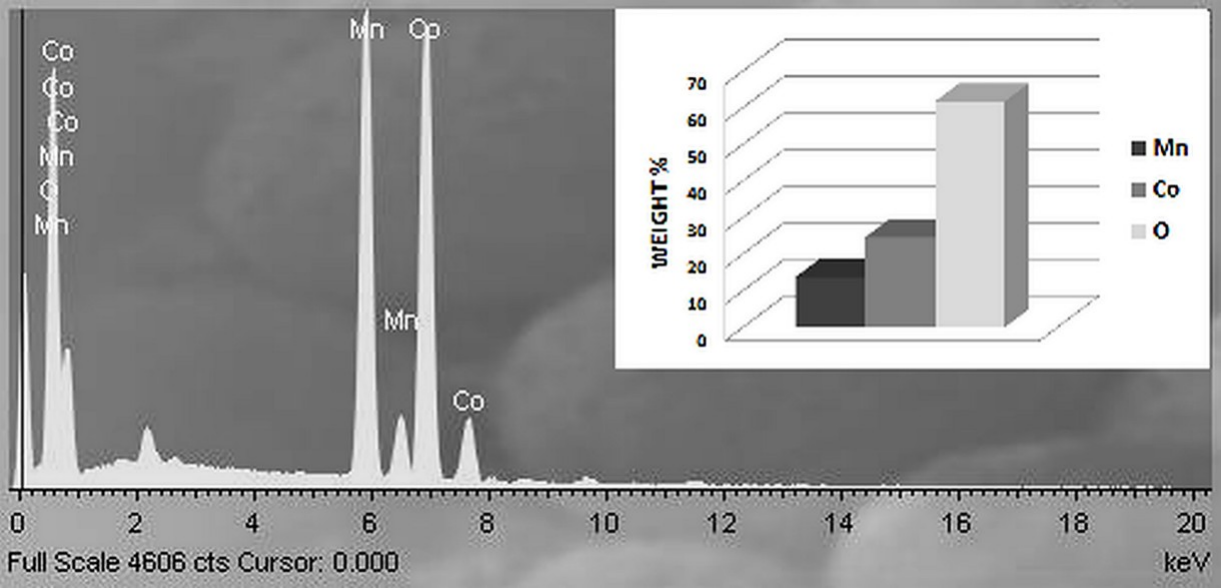
EDS spectrum for 0.5MnO-0.5Co_2_O_4_and element weight bar (%) diagram (13.7%, 24.58% and 61.72% for manganese, cobalt and oxygen, respectively).

Elemental composition analysis (Fig 5) of this porous spinel material obtained from energy dispersive spectrometer (EDS) further confirms the existence of manganese, cobalt and oxygen with weight percentage of 6.06%, 7.54% and 86.39%.

### Electrochemical performance of the GCE-MnCo_2_O_4_NPs

**Fig 6:**
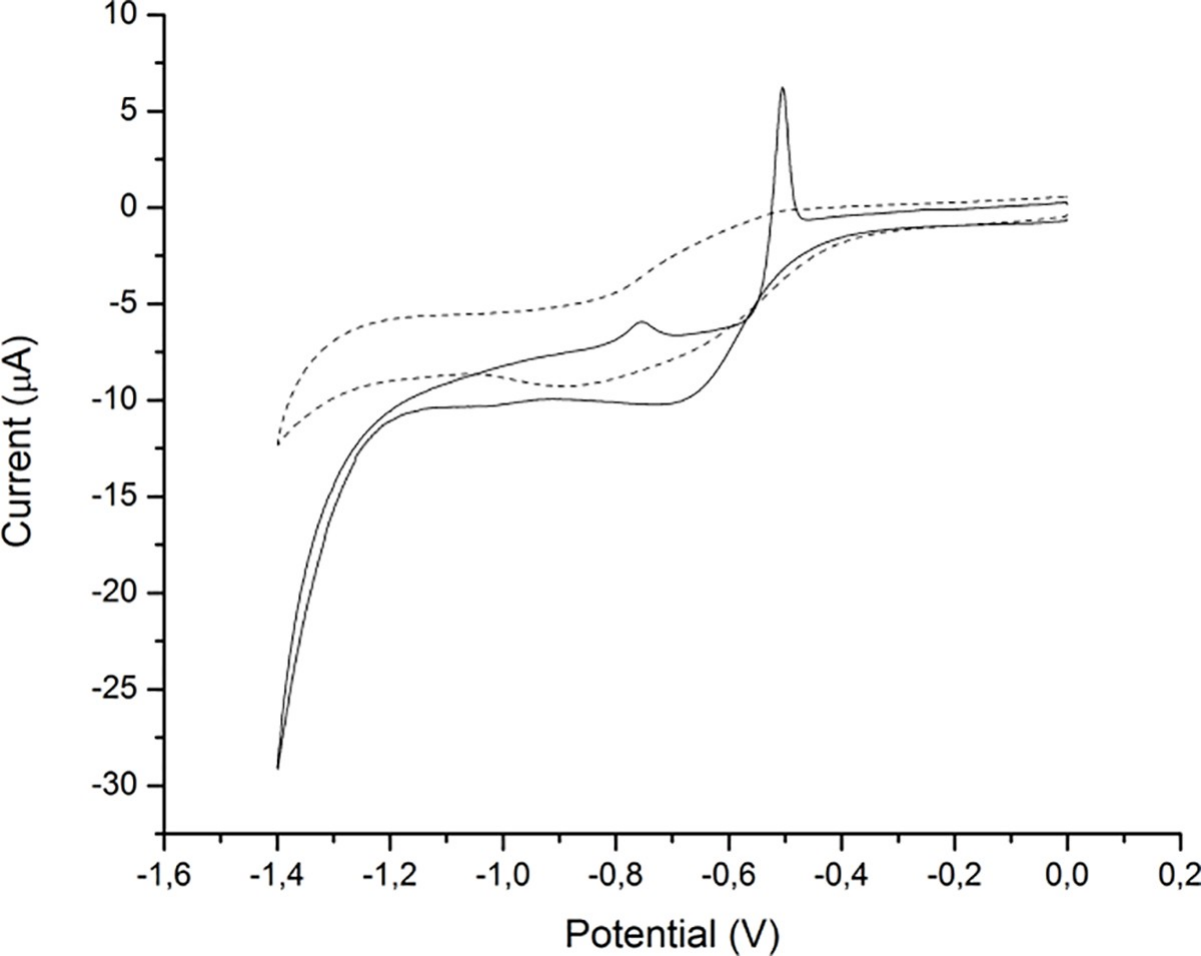
Cyclic voltammograms obtained on bare (dashed) and modified glassy carbon electrode (solid) for 10 μmol/dm^3^Pb(II) and Cd(II) at 150 mV/s scan rate.

Cyclic voltammograms of the sensor were recorded in the presence of 10 μmol/dm^3^Cd(II) and Pb(II) ions at a scan rate of 150 mV/s in H_2_SO_4_/KCl buffer (pH 2). Fig 6 shows voltammograms obtained for both bare and modified glassy carbon electrode. As it can be seen from the figure there are no visible signals on bare electrode whereas on the modified there are two well defined peaks at -500mV and -750 mV. The first peak was assigned to reduction of lead and second to cadmium, this finding was confirmed by recording cyclic voltammograms of solutions of individual ions (not shown here).

**Fig 7:**
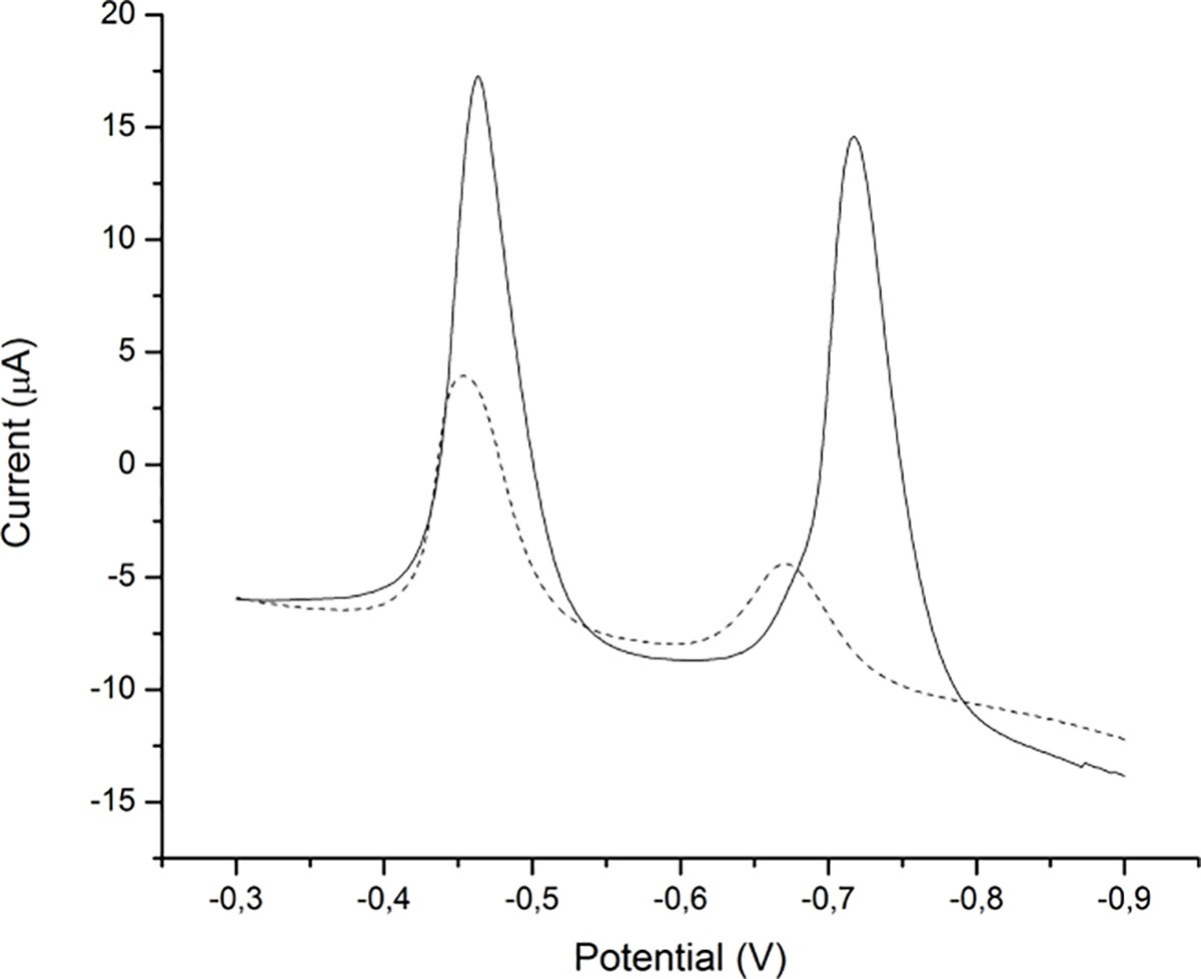
Comparison of LSAS voltammograms obtained for 1 μmol/dm^3^Pb(II) and Cd(II) on bare (dashed) and on modified GCE (solid).

Electrochemical performance of the sensor was investigated by linear sweep voltammetry (LSV). LSVs on both bare and GCE-MnCo_2_O_4_NPs show increased sensitivities for both analytes on modified electrode. Obtained peak currents for Pb(II) and Cd(II) on modified electrode were 24.7 and 25.4 μA, they are two times more sensitive for Pb(II) and six times more sensitive for Cd(II), obtained peak currents for bare electrode were 11.2 and 4.1 μA (Fig 7). The large difference in stripping potentials (250 mv) between the well defined peaks of Cd(II) and Pb(II) made simultaneous detection of these two ions in aqueous media by LSASV technique possible. Since the modified electrode has increased surface area due to the presence of the nanoparticles it has improved sensitivity toward the analytes. The larger surface area increase the number of active sites and it leads to higher signal-to-noise ratio [49].In order to achieve the best experimental conditions for determination of lead and cadmium content, the influence of pH, accumulation time and accumulation potential were examined.

### Effect of pH

**Fig 8:**
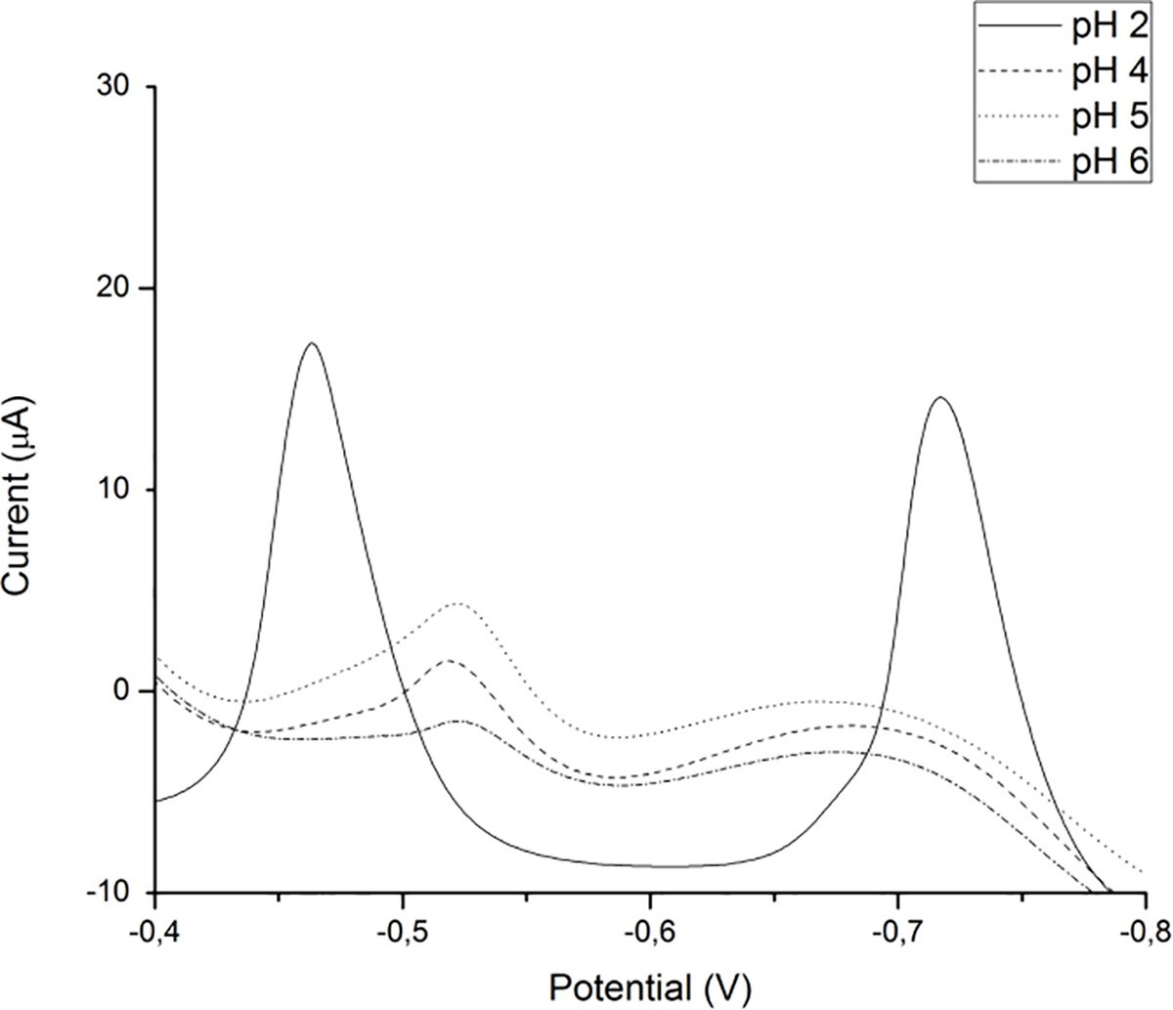
Effect of pH on Pb(II) and Cd(II) LSAS signals (concentration 1 μmol/dm^3^ for both Pb(II) and Cd(II)).

The effect of the pH value of the supporting electrolyte on the voltammetric response of Pb(II) and Cd(II) was examined in the pH range 2–6. In order to achieve the value pH 2 of the supporting electrolyte, a mixture of 20 mmol/dm^3^ sulfuric acid and 30 mmol/dm^3^ potassium chloride was used, as reported elsewhere [33] and for pH 4 to 6 range, the 0.1 mol/dm^3^ acetate buffer was used [50–52]. It is worth to mention that there are some discrepancies in literature concerning this issue. NiCo_2_O_4_ spinel structure showed lack of stability and electrochemical activity in acidic media [53] while synthesized cobalt ferrit spinel structure by hydrothermal technique confirmed stability of CoFe_2_O_4_ nanoparticles in a wide range of pH 2.2 to 10.8 [54]. From our experimental results a large, well defined and sharp current peaks, for both Pb(II) and Cd(II), appear in H_2_SO_4_/KCl buffer (pH 2), while acetate buffer (pH 4–6) gave less defined and intensive current peaks presented in Fig 8. The smallest concentration of Cd(II) that gives current peak in acetate buffer is 0.2 μmol/dm^3^ while in the H_2_SO_4_/KCl it is four times smaller.

**Fig 9:**
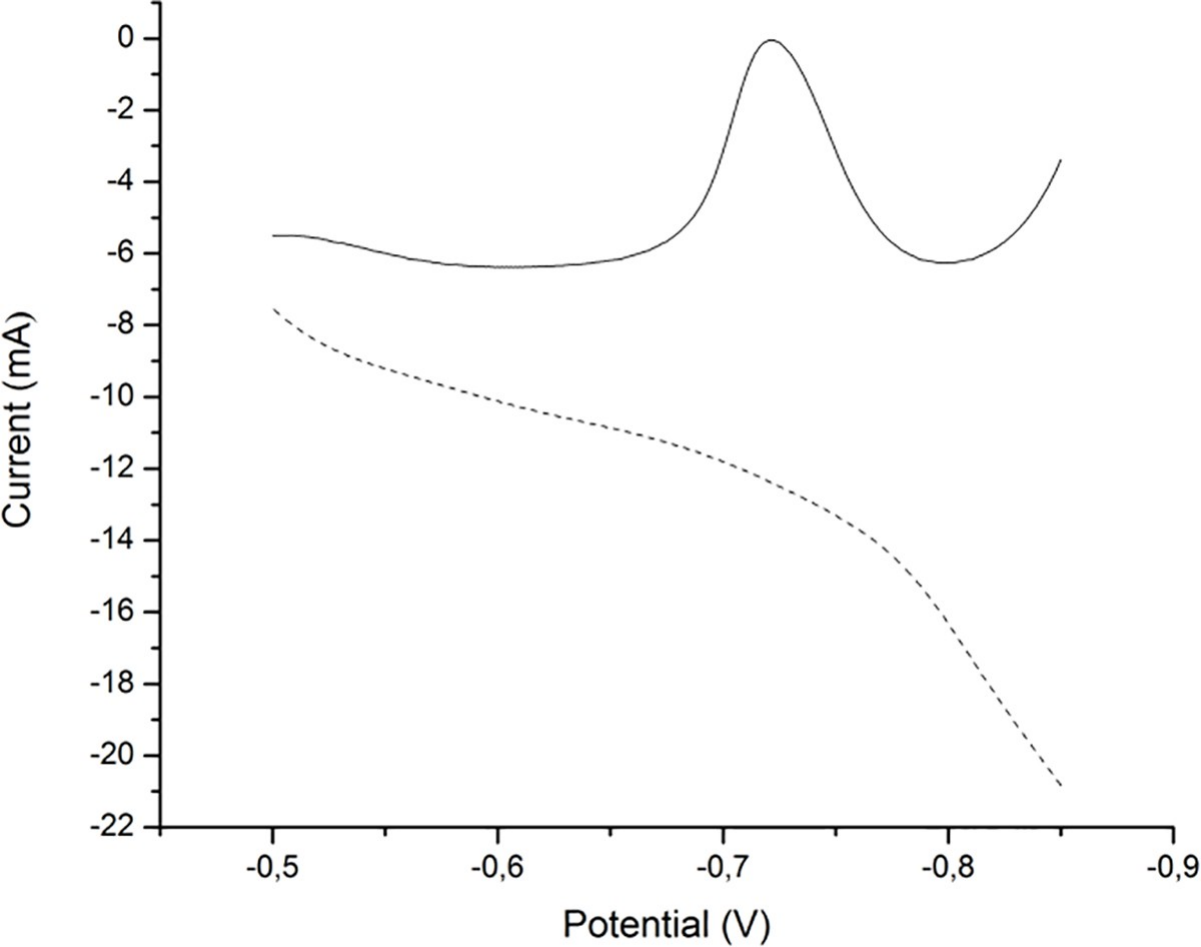
LSAS voltammogram obtained for 0.2 μmol/dm^3^Cd(II) in acetate buffer (pH 4) (dashed) and in H_2_SO_4_/KCl buffer, pH 2 (solid).

The Fig 9 shows barely visible peak of the of 0.2 μmol/dm^3^Cd(II) in acetate buffer (pH 4) while the same concentration in the H_2_SO_4_/KCl buffer gives a clear and much larger peak. This occurrence could be attributed to complex formation between metal ions and acetate buffer [55]. Electrochemical behavior of citrate-gel synthesized MnCo_2_O_4_ spinel material in H_2_SO_4_/KCl buffer is in a good agreement with observation for MnO_2_/carbon composites [33].

### Effect of accumulation time and potential

**Fig 10:**
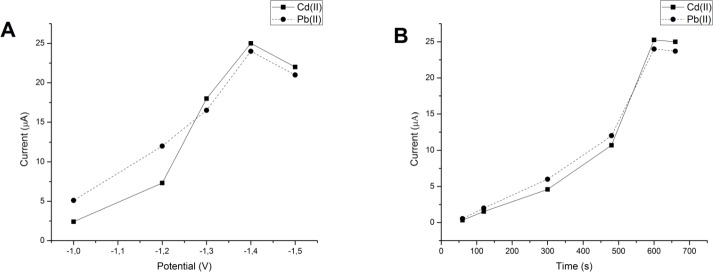
Dependance of oxidation current on accumulation potential (A) and time (B) of 1 μmol/dm^3^ Pb(II) and Cd(II).

The dependence of the linear sweep anodic stripping current peaks of Pb(II) and Cd(II) on the accumulation time and accumulation potential were examined. During deposition all solutions were stirred on a magnetic stirrer. As can be seen in Fig 10 accumulation potential and accumulation time have great influence on current intensities so they represent important steps in the optimization process. The effect of accumulation potential was investigated in the range from -1.0 to -1.5V, while the accumulation time was kept constant at 600 seconds. The highest peak was at potential -1.4 V and Fig 10A shows that even small potential change causes a significant increase of the oxidation current for both Pb(II) and Cd(II). Such behavior could be attributed to hydrogen adsorption onto surface of working electrode which was observed before [10,56]. Examination of accumulation time was investigated in the range 50–650 seconds at constant potential -1.4 V. As can be seen from the Fig 10B the current peaks for both Pb(II) and Cd(II) increase to 600 s and then become constant as is described previously [57]. Hence the optimal deposition conditions for simultaneous determination of Pb(II) and Cd(II) on GCE- MnCo_2_O_4_NPs were: accumulation potential of –1.4 V vs. Ag/AgCl, accumulation time of 600 s at pH 2 (H_2_SO_4_/HCl buffer).

### Interference study

In order to evaluate the selectivity of the proposed sensor GCE-MnCo_2_O_4_NPs, we examined the influence of some inorganic ions on the voltammetric response of Pb(II) and Cd(II). The experiment was performed by mixing solutions of common inorganic ions such as Mn(II), Zn(II), Cr(II), Hg(II), Cu(II) and Ni(II) with a solution containing 1.0 μmol/dm^3^ of Pb(II) and Cd(II). The ions present in water in abundant concentrations were also investigated K(I), Mg(II), and Ca(II). The concentration of potentially interfering ions was up to ten times higher than the concentration of Pb(II) and Cd(II) in solution. The obtained results are displayed in Table 1. As can be seen, the significant interference was only pronounced for copper ions. This interference can be related to migration and diffusion processes developing separately. It was reported previously that if sulfuric acid is used as a supporting electrolyte where copper sulfate is present as it is in our case, it can enhance conductivity and transference number of cupric ion [58]. Sulfuric acid also increases the diffusion and reduces the migration of copper ions, and this can be one of the reasons for significant interference of copper ions [58]. As previously suggested, this interference can be effectively overcome by the addition of Ferro-cyanide ions which creates a stable complex with Cu(II) and mask the copper ions [59].

**Table 1:** Interference study of some metal ions (10 μmol/dm^3^) on the voltammogram responses of 1 μmol/dm^3^Pb(II) and Cd(II). Supporting electrolyte: H_2_SO_4_/KCl (pH 2); deposition potential: -1.4 V, deposition time: 10 min; working electrode GCE-MnCo_2_O_4_NPs.

Intereferant	Peak current change (%)	
	Pb(II)	Cd(II)
**K(I)**	-3	-3
**Mg(II)**	+3	+2
**Ca(II)**	-1	-3
**Mn(II)**	-2	+1
**Zn(II)**	+6	+8
**Cu(II)**	-40	-36
**Hg(II)**	-7	-8
**Cr(III)**	-3	+1
**Ni(II)**	-3	-1

### Calibration data and application parameters

**Fig 11:**
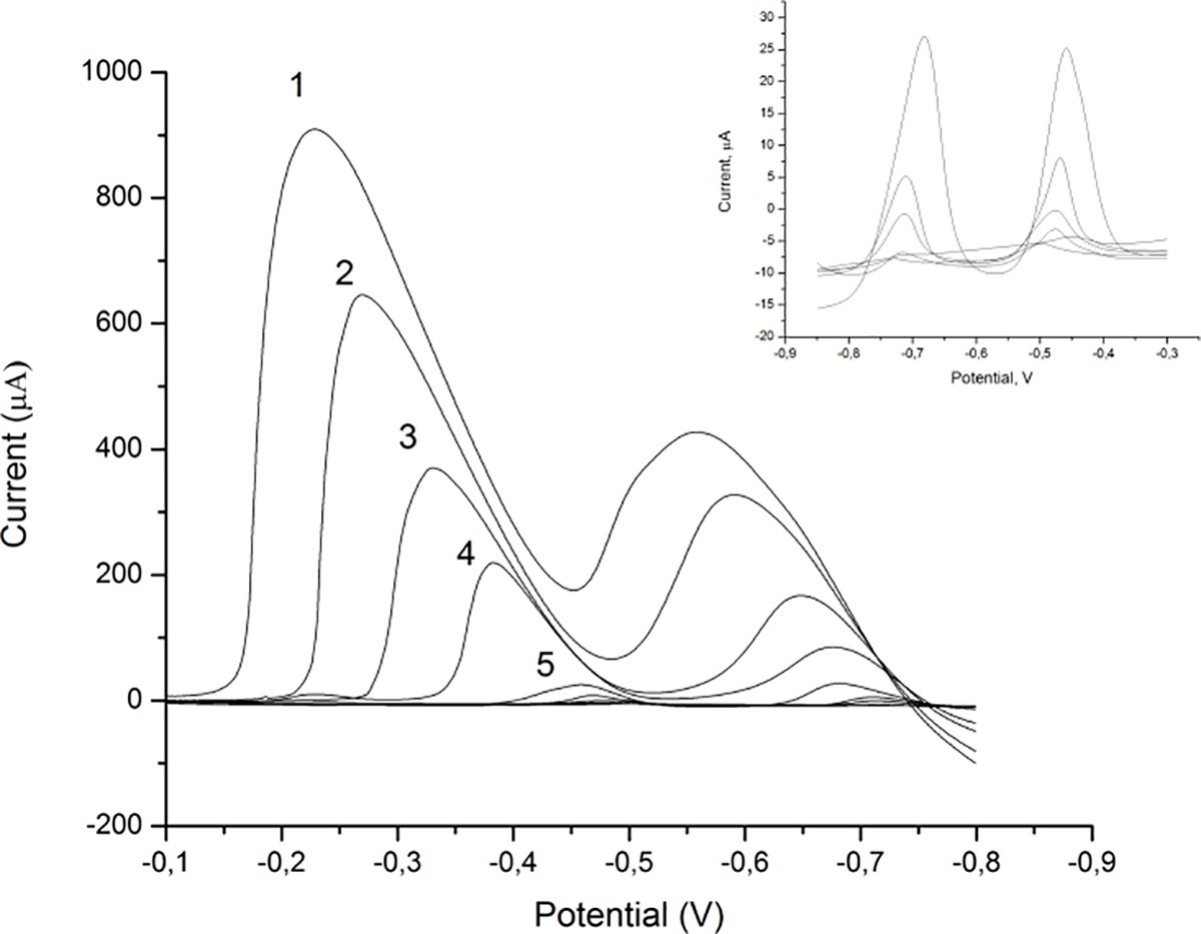
LSAS voltammograms obtained for Pb(II) and Cd(II) standard solutions for concentration range 1.6, 10, 20, 30 and 40 μmol/dm^3^, and the insert present voltammograms obtained for 0.05, 0.1, 0.2, 0.4, 0.8 and 1.6 μmol/dm^3^.

Under optimised parameters the linearity of the new sensor was investigated by recording of standard solutions of both analytes in the concentration range 0.05–40 μmol/dm^3^. The calibration curve and LSAS voltammograms for different concentration of Pb(II) and Cd(II) are presented in Fig 11. As seen from the Figure there is shift in peak potential with concentration of analytes. This phenomenon is observed in papers describing modification of GCE with various nanoparticles. In Fig 11, it can be seen that there is a slight displacement of the peak potentials, in case of Cd(II) only. This displacement can be attributed to overlapping of diffusion layers resulting from the stripping of metal (M^0^) from the surface of the electrode and the transition of metal ions (M^2+^) into the solution [57]. Linear ranges of the proposed method were from 0.05 to 40 μmol/dm^3^ for Pb(II) whereas oxidation current depends linearly on cadmium concentration in the ranges 0.05–1.6 and 1.6–40μmol/dm^3^.

**Fig 12:**
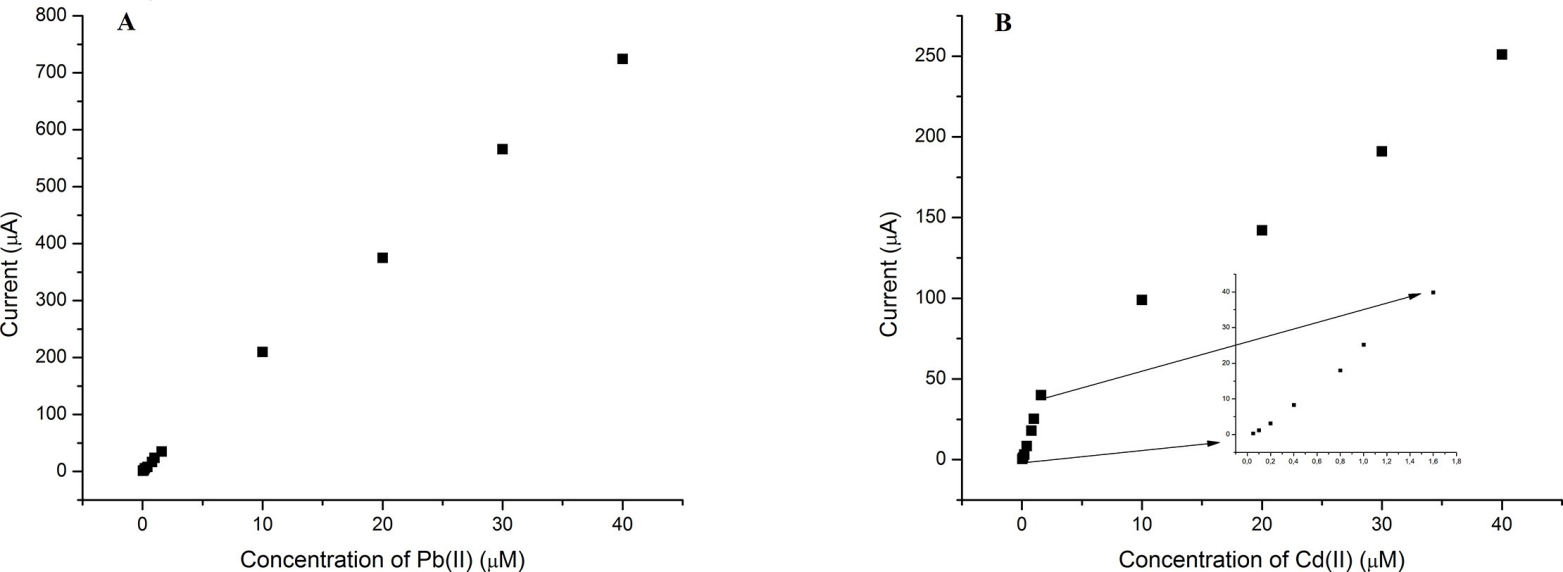
Calibration curves obtained for Pb(II) (A) and Cd(II) (B) in the concentration range 0.05–40 μmol/dm^3^. Insert on B figure is calibration curve for Cd(II) in the range 0.05–1.6 μmol/dm^3^.

Calibration curves (Fig 12) were described by linear equations, correlation coefficients and limits of detection. The corresponding equations were for Pb(II)—I (μA) = (18.3±0.2) c (μmol/dm^3^) + (5.2±0.3), correlation coefficient r^2^ = 0.9987, and detection limit (calculated as 3S/b, where S is standard deviation of the intercept and b is the slope) was 1.67 ppb for Pb(II) and I (μA) = (25.9±0.5)c (μmol/dm^3^)—(1.6±0.4), r^2^ = 0.9973 and corresponding detection limit 0.79 ppb for lower Cd(II) concentration range and I (μA) = (5.3±0.2)c (μmol/dm^3^) + (36.9±5.5), r^2^ = 0.9929 for higher Cd(II) concentration range. One of the advantages of this sensor, beside sensitivity is its reproducibility. It was evaluated under optimal conditions for five repetitive measurements of 0.05 μmol/dm^3^ for both Pb(II) and Cd(II) and corresponding relative standard deviations were 7.68% (Pb) and 3.46% (Cd). From presented results it can be concluded that this modification, MnCo_2_O_4_ particles, improves the sensitivity and the selectivity of the glassy carbon electrode. The sensor is easy to prepare, to use on cheap equipment and it analytical parameters are much better than some obtained by more sensitive electrochemical techniques (Table 2).

**Table 2:** Comparison of different modified electrodes for Pb(II) and Cd(II) determination.

Electrodes	Method	Pb(II)		Cd(II)		Ref.
		Linear range (μg/dm^3^)	Detection limit (μg/dm^3^)	Linear range (μg/dm^3^)	Detection limit (μg/dm^3^)	
**Nafion-Bi/NMC/GCE**	DPASV	0.5–100	0.05	2–200	1.5	[10]
**MnO**_**2**_**/GCE**	LSASV	-	5.58	-	5.84	[33]
**Cr**_**2**_**O**_**3**_**/CPE**	SWASV	10–800	3	10–800	3	[11]
**Cross-linkedchitosan/CNTPE**	LSASV	NA	NA	6.6–168.6	1.1	[12]
**AuNPs/CNFs**	SWASV	20.72–207.2	20.72	11.24–112.4	11.2	[52]
**SnO**_**2**_ **quantumdots**	CV	NA	NA	4990–44910	499	[61]
**BiNPs/SPCE**	SWASV	0–100	2	0–100	5	[50]
**Bi-film/CE**	SWASV	-	2.54	-	5.69	[62]
**SNAC/GCE**	DPASV	18.6–1181.0	1.2	10.1–539.6	2.7	[63]
**COOH-C4**	DPASV	280–2500	6.2	NA	-	[64]
**MnCo**_**2**_**O**_**4**_**/GCE (coprecipitationmethodsynthesis)**	DPSAV	NA	NA	2.3–120	0.72	[1]
**GCE-MnCo**_**2**_**O**_**4**_**NPs (citrate-gelcombustionmethod**	LSASV	10.4–8280	1.67	5.6–179.2	0.79	Thispaper

NMC/GCE: Nitrogendopedmicroporouscarbon/glassycarbonelectrode; CNTPE: Carbonnanotubepasteelectrode; AuNPs/CNFs: Aunanoparticles/carbonnanofibers; BiNPs/SPCE: Bismuthnanoparticles/screen-printedcarbonelectrode; CE:carbonelectrode; SNAC: sphericalcarbonnanoparticledecoratedactivatedcarbon; DPASV: differentialpulseanodicstrippingvoltammetry; SWASV: squarewaveanodicstrippingvoltammetry; NA: Notanalysed

**Fig 13:**
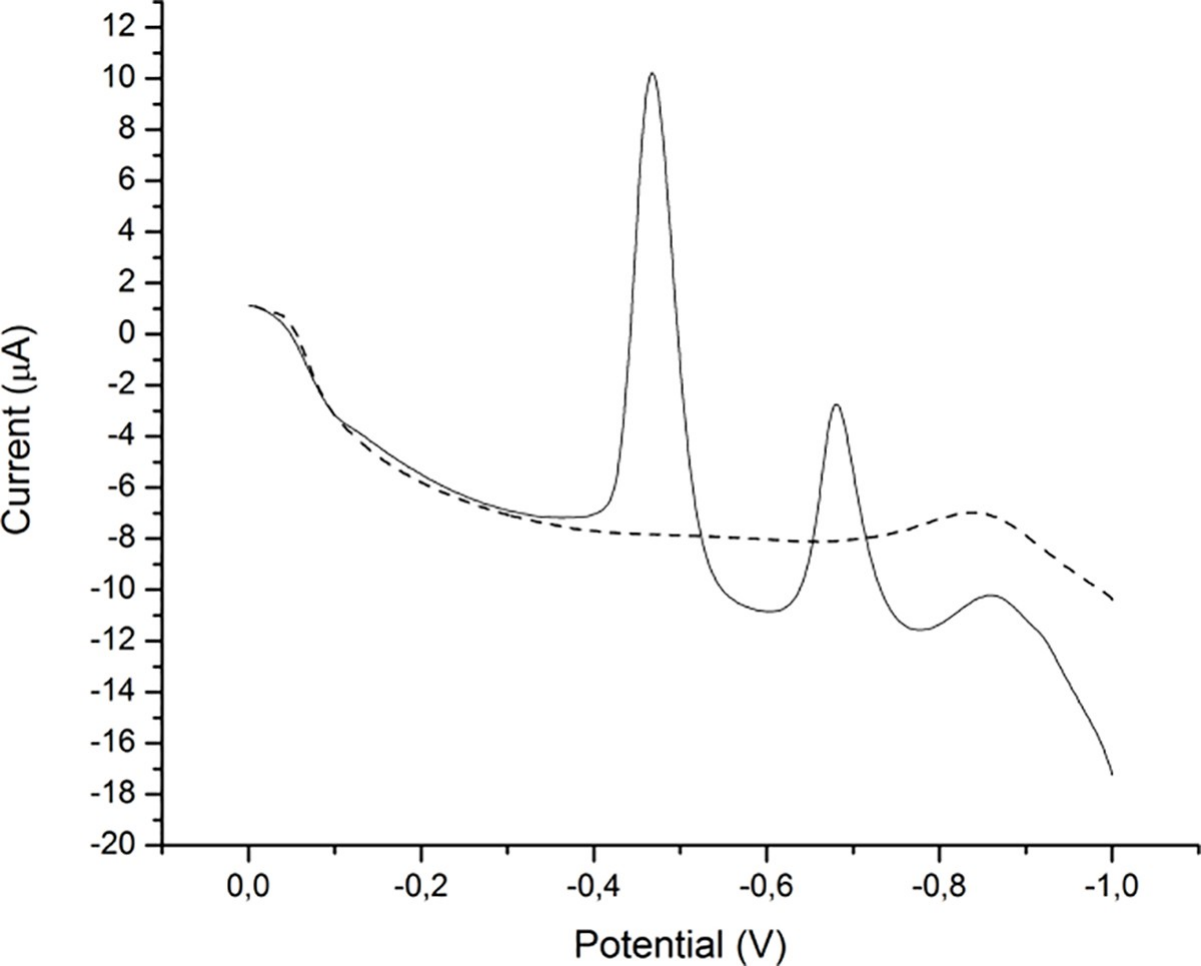
Voltammograms of river sample (dashed) and river sample spiked with 0.5 μmol/dm^3^Pb(II) and Cd(II) (solid).

The new sensor was applied on determination of analytes in real samples. Standard addition of 0.5 μmol/dm^3^ of both Pb(II) and Cd(II) caused increase of current at the sample potential and made the determination possible (Fig 13). The determination of lead and cadmium in three water samples are presented in Table 3. The results present average of three measurements, and the recovery is also calculated. The use of this sensor enabled determination of analytes in samples without complicated sample preparation. Also, these values are significantly lower than maximum allowed levels of lead and cadmium in drinking water given by the World Health Organization (10 μg/dm^3^ for Pb and 3 μg/dm^3^ for Cd) [60].

**Table 3:** Recovery tests for determination of Pb(II) and Cd(II) in water samples using GCE- MnCo_2_O_4_NPs (n.d.—not detectable; SD—standard deviation).

Sample	Analyte	Analyte in water before addition	Added (nmol/dm^3^)	Found (nmol/dm^3^)±SD	Recovery (%)
**Distilled** **water**	Pb(II)	n.d.	500.0	523.3±15.0	104.6
	Cd(II)	n.d.	500.0	504.2±22.1	100.8
**Tap** **water**	Pb(II)	n.d.	500.0	496.3±14.2	99.3
	Cd(II)	n.d.	500.0	487.5±21.4	97.5
**River** **water**	Pb(II)	n.d.	500.0	510.0±14.6	102
	Cd(II)	n.d.	500.0	548.0±24.1	109.6

## Conclusions

Porous MnCo_2_O_4_ spinel oxide was successfully synthesized by citrate-gel combustion technique. It was an interesting finding that this method of synthesis enabled simultaneous determination of two very toxic metals, lead and cadmium. The GCE-MnCo_2_O_4_NPs has shown excellent electrochemical properties such as fast current response, low detection limit and good selectivity due to unique structure. Under optimized conditions, the peak currents were increased with concentrations of metal ions linearly within two range 0.05–40 μmol/dm^3^ of Pb(II) and 0.05–1.6 and 1.6–40 μmol/dm^3^ of Cd(II). The detection limits for Pb(II) and Cd(II) were 1.67 and 0.79 μg/dm^3^, respectively. Additional advantages of this method for determination of cadmium and lead are easily, green and inexpensive synthesis of porous MnCo_2_O_4_ spinel and very simple fabrication of modified GC electrode. The sensor was successfully validated on real water samples, therefore, it can be easily used as portable field device for onsite control of heavy metal concentration.
